# Supplementary material for: Effect of Molecular Weight and Degree of Substitution on the Physical-Chemical Properties of Methylcellulose-Starch Nanocrystal Nanocomposite Films
Source: Polymers (Basel). 2021 Sep 27;13(19):3291. doi: 10.3390/polym13193291 (PMC8513015; doi:10.3390/polym13193291)
Supplement: Supplementary file 1 [file polymers-13-03291-s001.zip › polymers-1387232-supplementary.pdf]

Effect of Molecular Weight and Degree of Substitution on the Physical-Chemical Properties of Methylcellulose-Starch Nanocrystal Nanocomposite Films

Qian Xiao [1], Min Huang [1], Xiaolan Zhou [1], Miaoqi Dai [1], Zhengtao Zhao [2] and Hui Zhou [1,*]

[1] School of Food Science and Technology, Hunan Agricultural University, 410128 Hunan, China; qianxiao@hunau.edu.cn (Q.X.); minhuang11@126.com (M.H.); XiaolanZ90@126.com (X.Z.); MiaoqiD98@163.com (M.D.)
[2] Department of Food Science, University of Guelph, Guelph, ON N1G 2W1, Canada; zhengtaozhao85@gmail.com
* Correspondence: zhouhui@hunau.edu.cn; Tel.: +86-0731-8461-7013

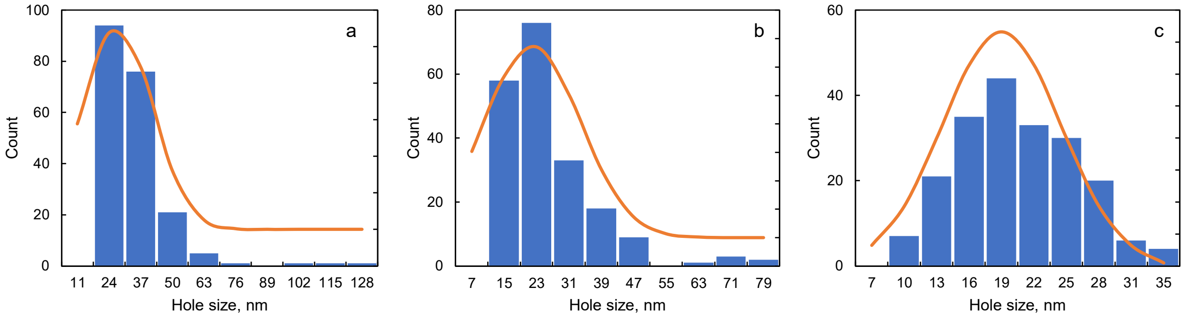

Figure S1. Histograms showing the distribution of hole size of MC film: (**a**) M20; (**b**) A4C; (**c**) A4M.
